# Importance of Prevention and Control of Nosocomial Infections in Iran

**Published:** 2018-02

**Authors:** Salman KHAZAEI, Somayeh KHAZAEI, Erfan AYUBI

**Affiliations:** 1. Dept. of Epidemiology, School of Public Health, Hamadan University of Medical Sciences, Hamadan, Iran; 2. Dept. of Nursing, School of Nursing and Midwifery, Rafsanjan University of Medical Sciences, Rafsanjan, Iran; 3. Dept. of Epidemiology, School of Public Health, Shahid Beheshti University of Medical Sciences, Tehran, Iran

## Dear Editor-in-Chief

Nosocomial infection (NI) is considered as a hospital-acquired infection by a patient admitted to hospital for other reason than mentioned infection. Otherwise, an infection occurred in hospital and at the time of admission, the patient did not have the infection and also was not in the incubation period (1). Reducing the level of patient immunity; the increasing variety of medical techniques and invasive procedures creates potentially paths of infection, transmission of resistant to treatment bacteria, and poor infection control practices can promote infection among hospitalized patients. 8.7% of patients admitted to hospital had NI. More than 1.4 million people worldwide at any time are engaged in NI (2). The rates of infection are higher among patients with high susceptibility for example old age, underlying disease, or receiving chemotherapy (3).

According National Nosocomial Infections Surveillance (NNIS), reported from 44 medical universities in Iran in 2011, from 5249877 hospitalized patients for 38604 patients had occurred NI (mean incidence rate: 0.74%, range from 0.05% in Zabol to 2.66% in Tehran). 54.6% of them were male (Table 1).

**Table 1:** Frequency and percent of common nosocomial infections in Iran, 2011

***Nosocomial infection type***	***Frequency***	***Percent***
Urinary tract Infection	10343	26.8
Respiratory infection	9937	25.7
Surgical Infection	6111	15.8
Blood infection	5437	14.1
Other	6776	17.6
Total	38604	100

NI imposed defect in function and increased stress for patients and are one of the major causes of death (4). The economic costs of these infections are notable due to prolonged stay in hospital and indirect costs, for example, lost work or job, increased drugs consumption, necessity of isolation, and the use of extra laboratory or other diagnostic tests also added to costs (5, 6). Length of inpatient is a major problem, for patients with surgical wound infections about 8.2 d increased duration of hospitalization, that it is fluctuating from 3 d for gynecology to about 10 d for general surgeries and 19.8 for orthopedic surgeries (7).

The considerable incidence of NI among patients, for prevention of NI, each one should work responsibly to reduce the risk of transmission of infection to patients and other staff, this can achieve with providing personal protective equipment for staff, management, preparing materials and products, and education and training of health workers periodically (8).
